# Effectiveness of Peng’s Shengjing recipe on male asthenospermia caused by kidney yang deficiency: A randomized pilot study

**DOI:** 10.15537/smj.2023.44.3.20220676

**Published:** 2023-03

**Authors:** Guangyao Xu, Yu Peng, Chunyan Chen

**Affiliations:** *From the Eighth Department of Surgery (Xu), The Shanghai Municipal Hospital of Traditional Chinese Medicine; from Urology Surgery (Peng), Yueyang Hospital of Integrated Traditional Chinese and Western Medicine; and from the Shanghai Research Institute of Qigong (Chen), Taiji Health Center, Shanghai University of Traditional Chinese Medicine, Shanghai, China.*

**Keywords:** asthenospermia, deficiency of kidney yang, Peng’s Shengjing recipe, traditional Chinese medicine

## Abstract

**Objectives:**

To examined the efficacy and safety of Peng’s Shengjing recipe in treating asthenospermia with deficiency and failure of kidney yang. The traditional Chinese medicine (TCM) Peng’s Shengjing recipe might have benefits in treating male asthenospermia.

**Methods:**

This randomized, positive drug-controlled, single-blind pilot study enrolled outpatients from the Third Department of Traditional Chinese Medicine Surgery, Shanghai University of Traditional Chinese Medicine, Shanghai, China, between April 2020 and September 2020. A total of 99 participants were randomized to Shengjing recipe (n=50) and Xuanju capsule (n=49). They were treated for 12 weeks. The primary endpoint was routine semen examinations, including the percentage of sperm motility rated grade A, A+B, and A+B+C, and the clinical effective rate. The secondary endpoints were the levels of gonadotropins.

**Results:**

The A grade sperms (18.9% versus [vs.] 13.9%, *p*=0.030) and A+B grade sperms (42.9% vs. 32.7%, *p*<0.001) were higher in the Shengjing recipe group than the Xuanju capsule group. The effective rates were 68% and 53.1% in the Shengjing recipe and Xuanju capsule groups (*p*=0.128). No safety signals were observed.

**Conclusion:**

Peng’s Shengjing recipe improves the quality of sperms and is effective in treating clinical asthenospermia of deficiency of kidney yang. The treatment was well tolerated, without obvious hepatorenal toxicity.

**Chinese Clinical Research Registry No.: ChiCTR2000030845**

**T**he worldwide incidence of infertility for reproductive-aged couples is approximately 15%.^1,2^ In the United States, male factor infertility may affect approximately 9% of men aged 15-44. Male factors alone are involved in approximately 30% of infertile couples.^3^ In Western countries, the interventions for managing male infertility mainly aim to improve semen quality. The interventions included changes in lifestyle habits (changes in diet, smoking cessation, reducing alcohol exposure, exercise, weight loss, avoiding lubricants, and avoiding increased scrotal temperature), antioxidant supplementation, hormones or antihormones to correct hormonal imbalances, varicocele repair, and sperm retrieval for in vitro fertilization.^1,4^ Still, these methods cannot achieve fertility in all patients.^1^

Asthenospermia refers to decreased sperm count and motility.^1^ In China, Liu et al^5^ and Huang et al^6^ showed that semen quality declined in sperm donors in the past 2 decades. Male infertility corresponds to the category of “childless, cold sperm, seed, and not male” in traditional Chinese medicine (TCM).^7,8^ If kidney yang is insufficient, the kidney loses warmth, semen becomes thin, the sperm count decreases, and the sperm activity is low, resulting in asthenospermia.^9,10^ Therefore, the TCM treatment of male infertility should focus on warming and tonifying kidney yang, enriching kidney essence, and fully stimulating the vitality of semen.^7,8^

Peng’s Shengjing recipe is mainly composed of the Mantis Egg-case, hive, rigid silkworm, burnt astragalus, codonopsis, atractylodes, rehmannia, cornus, and Yizhiren.^11^ The above drugs were used together to enhance the effect of sperm production. Sun et al^11^ showed that Peng’s Shengjing recipe could improve the parameters of asthenospermia. Basic experiments suggested that this effect might be related to the up-regulation of gonadotropins, including follicular-stimulating hormone (FSH) and testosterone (T).^11^ Research is necessary to determine the benefits of Peng’s Shengjing recipe in treating male asthenospermia.

Therefore, this study aimed to examine the effectiveness and safety of Peng’s Shengjing recipe in the treatment of male asthenospermia with kidney yang deficiency. The results could help provide more treatment options for asthenospermia patients with kidney yang deficiency.

## Methods

The related studies on the subject was found through a search in PubMed, Embase, the Cochrane Library, and CKNI. Google was used to search the grey literature.

This randomized, positive drug-controlled, single-blind pilot study enrolled outpatients from the Third Department of Traditional Chinese Medicine Surgery, Shanghai University of Traditional Chinese Medicine, Shanghai, China, between April and September 2020. The study was approved by the Ethics Committee of the Shanghai Municipal Hospital of Traditional Chinese Medicine, Shanghai, China (approval number: 2018SHL-KYYS-16). All patients provided written informed consent before any study procedure. The work was carried out in accordance with the Institutional Review Board and carried out with the ethical standards set forth in the 1975 Helsinki Declaration.

The inclusion criteria were: i) 23-40 (inclusive) year-old male patient; ii) diagnosed with male infertility according to the diagnostic criteria of Western medicine;^1^ iii) met the classification standards of TCM for male infertility;^8^ iv) voluntarily participation; and v) no use of drugs related to male infertility within 30 days before treatment. The exclusion criteria were: i) azoospermia; ii) those with retrograde sperm drainage; iii) taking anti-epileptic or anti-tumor drugs that hinder sperm production and sperm motility; iv) congenital malformations, obstruction of the spermatic pathway, or testicular atrophy; v) immune infertility or varicocele; vi) orchitis; vii) epididymitis; viii) moderate and severe prostatitis; ix) genital surgery history; x) history of trauma that might affect fertility; xi) cardiovascular, liver, kidney, hematopoietic system, endocrine, immune, or genetic diseases; or xii) patients with mental diseases.

Asthenospermia due to kidney yang deficiency syndrome was defined as a loss of libido, low sperm count, low survival rate, weak motility, or poor ejaculation. The waist and knees are sore, tired, and weak, and the urine is long and clear. The tongue is pale, the coating is thin and white, and the pulse is heavy and thin.^8^

The dropout criteria were: i) participants with poor compliance (those who failed to follow the treatment as prescribed or use other treatment methods for male asthenospermia during the study) and ii) participants with serious adverse events (SAEs) during the study treatment.

The randomization sequence was prepared by a third-party statistician using a random number table and in the form of sealed sequential envelopes. The patient received the indicated treatment according to the opened envelopes. Only the patients were blind to grouping.

Peng’s Shengjing recipe was prepared using 18 grams (g) of Mantis egg case, 18 g of honeycomb, 18 g of rigid silkworm, 9 g of roasted astragalus, 9 g of codonopsis, 15 g of atractylodes, 15 g of rehmannia, 15 g of cornus, and 9 g of Yizhiren. The participants had to take 200 ml orally twice a day. A course of treatment was 12 weeks.

The control group received the compound Xuanju capsule (Z20060462, Zhejiang Shiqiang Pharmaceutical Co., Ltd., 0.42 g/capsule) 3 times a day, 3 capsules each time, orally. Previous studies showed that Xuanju capsules could increase sperm density, sperm motility, and sperm activity and reduce the rate of sperm deformity without obvious adverse events.^9,12,13^

The percentage of sperms rated grade A, A+B, and A+B+C, semen volume, and efficacy were recorded by routine semen examinations. Each semen collection was carried out by masturbation after 3-5 days of abstinence. Semen quality was evaluated using computer-aided, fully automatic semen analysis (CASA) technology. The items included sperm motility and other parameters according to international standards.^14^

Treatment efficacy was rated as 1-markedly effective (sperm motility was normal after treatment), 2-effective (improvement of sperm motility to >30% for grade A+B and clinical symptoms improved), or 3-invalid (sperm motility <30% for grade A or A+B or no change after treatment. The clinical effective rate was calculated as (significantly effective number + effective number) / total number of people treated × 100%. The levels of gonadotropins (FSH, luteinizing hormone [LH], and testosterone) in the 2 groups were recorded before and after treatment.

According to the World Health Organization (WHO) criteria, asthenospermia was defined as sperm motility rated <25% for class A or rated <50% for class A+B.^14^ The primary endpoint was the results of routine semen examinations, including the percentage of sperm motility rated grade A, A+B, and A+B+C. The secondary endpoints were the clinical effective rate and the levels of gonadotropins (FSH, LH, and testosterone). The safety assessment indexes (creatinine, alanine transaminase (ALT), and aspartate transaminase (AST) were examined before and after treatment. The participants were followed up for 3 months after treatment by an attending surgeon with 10 years of experience in male infertility.

According to preliminary data, the clinical effective rate of Peng’s Shengjing recipe was approximately 60%, while the clinical effective rate of Fufang Xuanju capsule was approximately 40%. Therefore, the number of cases in each observation group was set as 30 cases/60%=50 cases.

### Statistical analysis

The data were analyzed using the Statsitical Package for the Social Sciences, version 16.0 (SPSS Inc., Chicago, USA). The continuous variables with a normal distribution were presented as means ± standard deviations and analyzed using the independent-samples t-test (2-group comparisons). Otherwise, they were presented as medians (ranges) and analyzed using the Mann-Whitney U test. Continuous data from repeated measurements were statistically analyzed using repeated measurements analysis of variance. Categorical data were presented as numbers and precentages (%); they were analyzed using the Chi-square test. *P*-values of <0.05 were deemed statistically significant.

## Results

**Figure 1:**
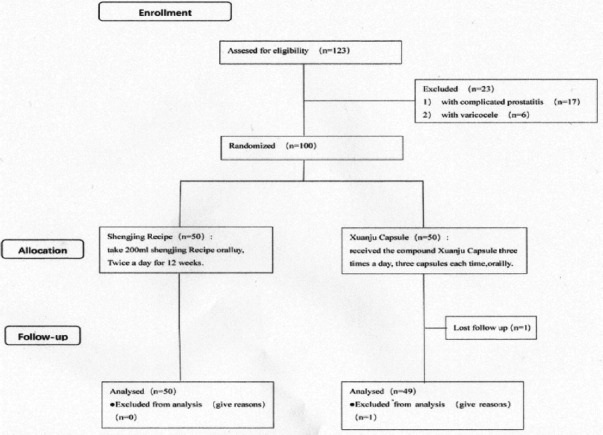
- Patient flow chart.

A total of 123 outpatients were enrolled in this study from April to September 2020. Twenty-three participants were excluded due to complicated prostatitis (n=17) or varicocele (n=6). Therefore, 100 participants were randomized to Shengjing recipe (n=50) and Xuanju capsule (control group: n=50; Figure 1). One participant in the control group was lost to follow-up. Finally, 99 participants entered the analysis. Table 1 shows that the 2 groups were comparable in terms of grade A and A+B+C sperms, semen volume, FSH, testosterone, creatinine, and AST (all *p*>0.05), but the Shengjing recipe group had lower proportions of A+B grade sperms (21.3% versus [vs.] 28.9%, *p*=0.006), lower LH levels (4.09 vs. 5.5 mIU/mL, *p*=0.038), and lower ALT levels (21.1 vs. 32.09 IU/L, *p*<0.001).

**Table 1:** - Characteristics of the patients (N=123).

Characteristic	Shengjing recipe (n=50)	Xuanju capsule (n=49)	*P*-value
Age (years), mean±SD	35.31±3.18	34.52±4.20	0.920
Body mass index (BMI, kg/m^2^), mean±SD	21.51±1.47	21.39±1.23	0.775
Smoking, n (%)	14 (28.0)	14(28.5)	0.963
Alcohol drinking, n (%)	11 (22.0)	10 (20.4)	0.805
Duration of asthenospermia (years), mean±SD	3.77±1.26	3.81±1.44	0.658
***Sperm motility class, %, median [range]***			
A	8.49 (6.0), [10.81]	8.3 (3.4), [14.88]	0.883
A+B	21.28 (16.3), [28.6]	28.85 (18.5), [40.67]	0.006
A+B+C	50.76 (31.3), [71.98]	48.65 (35.7), [70.98]	0.475
Semen volume, ml, mean±SD	3.24±1.33	2.98±1.73	0.313
***Gonadotropins, mean±SD***			
FSH, mIU/mL	4.46±1.90	3.73±2.47	0.103
LH, mIU/mL	4.09 (2.6), [5.65]	5.5 (3.2), [7.46]	0.038
Testosterone, nmol/L	15.5 (13.9), [17.43]	14.8 (13.2), [16.06]	0.168
***Liver and kidney function***			
Creatinine, µmol/L	64.6 (53.8), [78.28]	73.5 (55.5), [84.34]	0.155
ALT, IU/L	21.1 (14.1), [27.5]	32.1 (26.1), [38.4]	<0.001
AST, IU/L	35.1 (29.6), [38.6]	36.3 (26.1), [42.9]	0.834
*P*<0.05 was considered statistically significant. Grade A sperms are fast progressive sperms that swim quickly in a straightforward direction. Grade B sperms are slow progressive sperms that move forward but in a haphazard line or maybe a little slower. FSH: follicular-stimulating hormone; LH: luteinizing hormone, ALT: alanine transaminase, AST: aspartate transaminase, SD: standard deviation			

After 12 weeks of treatment, in both groups, the sperm quality (grade A sperm and grade A+B sperm) had improved (all within-group *p*<0.05). The proportion of grade A and A+B sperms were higher in the Shengjing recipe group than in controls (A: 18.9% vs. 13.9%, *p*=0.030; A+B: 42.9% vs. 32.7%, *p*<0.001). The effective rates were 68% and 53.1% in the Shengjing recipe and control groups (*p*=0.128) (Table 2).

**Table 2:** - Effectiveness and safety assessments (N=123).

Indexes	Shengjing recipe (n=50)		P_time_	Xuanju capsule (n=49)		P_time_	P_group_	P_time×group_
	Baseline	After intervention		Baseline	After intervention			
***Sperm motility class, %, median (range)***							
A	8.5 (6.0), [10.81]	18.9 (9.8), [26.30]	<0.001	8.3 (3.4), [14.88]	13.9 (7.7), [19.42]	0.001	0.030	/
A+B	21.3 (16.3) [28.60]	42.9 (34.8), [50.26]	<0.001	28.9 (18.5), [40.67]	32.7 (25.9), [40.14]	0.047	<0.001	/
A+B+C	50.8 (31.3), [71.98]	66.2 (48.5), [80.22]	0.037	48.7 (35.7), [70.98]	70.1 (38.8), [86.78]	0.022	0.758	/
***Effectiveness measurement***							
Markedly effective, n	/	19	/	/	10	/	0.128	/
Effective, n	/	15	/	/	16	/		/
Invalid, n	/	16	/	/	23	/		/
Clinical effective rate, %	/	68.0	/	/	53.1	/		/
Semen volume, ml, mean±SD	3.24±1.33	3.44±1.79	0.398	2.98±1.73	3.21±1.56	0.321	0.645	/
FSH, mIU/Ml	4.46±1.90	6.42±2.07	/	3.73±2.47	5.50±2.39	<0.001	0.015	0.752
LH, mIU/mL	4.21±2.36	5.65±2.4	/	5.29±2.43	5.23±2.14	0.031	0.347	0.019
Testosterone, nmol/L	15.33±2.38	20.22±2.14	/	14.81±2.24	15.93±2.06	<0.001	<0.001	<0.001
Creatinine, µmol/L	65.27±15.37	66.27±25.51	/	69.66±18.46	71.18±20.44	0.672	0.099	0.931
ALT, IU/L	21.73±9.6	25.11±11.47	/	31.79±9.75	28.46±16.43	0.987	<0.001	0.066
AST, IU/L	34.46±10.01	29.64±11.55	/	34.21±10.49	36.84±11.15	0.481	0.025	0.018
FSH: follicular-stimulating hormone, LH: luteinizing hormone; ALT: alanine transaminase, AST: aspartate transaminase. *P*<0.05 was considered statistically significant.								

In the Shengjing recipe group, FSH (4.46±1.90 vs. 6.42±2.07mIU/Ml, *p*<0.001), LH (4.21±2.36 vs. 5.65±2.4 mIU/mL, *p*=0.031), and testosterone (15.33±2.38 vs. 20.22±2.14 nmol/L, *p*<0.001) increased after the intervention. The FSH (6.42±2.07 vs. 5.50±2.39 mIU/Ml, *p*=0.015) and testosterone (20.22±2.14 vs. 15.93±2.06 nmol/L, *p*<0.001) were higher in the Shengjing recipe group compared with the control group. Time×group interactions were observed for LH (P_time×group_=0.019) and testosterone (*p*_time×group_<0.001) but not for FSH (*p*_time×group_=0.752) (Table 2).

The liver and kidney functions (blood creatinine, ALT, and AST) were not significantly different between the 2 groups (all *p*>0.05). The Peng’s Shengjing recipe group showed lower ALT (25.11±11.47 vs. 28.46±16.43 IU/L, *p*<0.001) and AST (29.64±11.55 vs. 36.84±11.15 IU/L, *p*=0.025) levels than the control group (Table 2). One patient in Peng’s Shengjing recipe group had epigastric discomfort after oral administration on an empty stomach, but it was relieved by itself. One patient in the control group had frequent loose stools, but it was relieved by itself. No patients discontinued treatment or had dose reduction during the whole study period.

## Discussion

The TCM Peng’s Shengjing recipe might have benefits in treating male asthenospermia. This study suggests that Peng’s Shengjing recipe improves the proportions of grade A and grade A+B sperms in patients with clinical asthenospermia due to kidney yang deficiency and increases the FSH and T levels but shows lower ALT and AST levels than the control group (Xuanju capsule).

In Peng’s Shengjing recipe, the mantis egg case consolidates the essence and reduces urine, strengthens the kidney yang but not the heat, replenishes Qi and blood without affecting the diaphragm, and has the function of adjusting the dysfunction of multiple organs.^15^ Various TCM preparations strengthen the kidney and help yang use stiff silkworm, roasted astragalus, codonopsis, and cooked rehmannia as adjuvant drugs.^15^ Among them, stiff silkworm dispels wind and relieves spasms, resolves phlegm, and dispels congestion. Roasted astragalus and codonopsis have the functions of nourishing Qi and strengthening the kidney and spleen. Cooked rehmannia nourishes the blood and yin and fills the essence and marrow.^15^ Atractylodes macrocephala, common macrocarpium fruit, and Fructus alpiniae oxyphyllae are used as adjuvants. Atractylodes macrocephala invigorates the spleen and replenishes Qi. Common macrocarpium fruit tonifies the liver and kidney, astringes essence, and stops sweating. Fructus alpiniae oxyphyllae warms the kidney and consolidates the essence. The above drugs were used together to enhance the effect of sperm production.^11^

In the present study, Xuanju capsule, a classical TCM for male infertility, was used as positive control and showed improvements in grade A and A+B sperms.^9,12,13^ Shengjing recipe also improved grade A and A+B sperms, and to a greater extent than in the Xuanju capsule group. These results are supported by Sun et al,^11^ who showed improvements in sperm motility, concentration, and morphology after 12 weeks of treatment with Shengjing recipe in patients with oligoasthenospermia. Yang et al^16^ showed that Shengjing recipe could improve sperm density and motility, and increase the pregnancy rate. Similar results were observed by Song et al,^17^ Guo et al,^18^ Zhang et al,^19^ and Chen et al.^20^

These effects are also supported by animal studies that shed some light on the mechanisms of Shengjing recipe. Indeed, animal studies reported increased sperm quality and quantity when treated with the Shengjing recipe, including animal models of drug-induced asthenospermia.^8,21-23^ Yang et al^24^ showed that Shengjing recipe increased the production of nitric oxide in the rat testicles leading to an enhanced anti-oxidation power that is protective for the sperm. In addition, Shengjing recipe upregulates integrin α6/β1 expression through the PI3K/AKT pathway.^25^ Integrin α6/β1 is a determinant of sperm function.^26^

In the present study, Shengjing recipe increased the levels of FSH and T, but the exact clinical significance of these changes is unknown since the values were within the normal values at baseline and after treatment. Still, these changes were also observed in previous studies. Sun et al^11^ showed that Shengjing recipe could increase FSH and T levels. Chen et al^20^ showed that Shengjing recipe could increase FSH, LH, and T.

This study did not identify any safety signals using the Shengjing recipe. The kidney and liver parameters remained in the normal range. No obvious hepatorenal toxicity was recorded. This observation is supported by other studies of Shengjing recipe.^11,16-20^ The Shengjing recipe group had lower ALT and LH levels at baseline compared with the control group, but these differences disappeared after 12 weeks of treatment, and the ALT levels of all patients remained within the limits of normal. Hence, these results suggest that Peng’s Shengjing recipe is safe.

### Study limitations

The study population was relatively small, but it complied with the initial power calculation. No placebo control group was included, preventing the observation of the absolute effect of the Shengjing recipe. The effect could only be observed relative to Xuanju capsule. An untreated group could not be included for ethical reasons because asthenospermia is a treatable condition using TCM and because all included participants were seeking fertility treatments. The pregnancy outcomes were not evaluated, but the present study was not powered to do so. The blood and semen parameters were limited, impairing a complete analysis of the beneficial effects of the Shengjing recipe. In TCM clinical practice, sperm concentration and normal sperm form are closely related to the traditional concept of “body fluid”. In addition, sperm viability and hormone levels in this study are related to the TCM concept of “kidney yang”, and the Chinese medicine prescription selected in this study was mainly for the treatment of patients with kidney yang deficiency. Therefore, sperm concentration and normal sperm morphology were not included in the observation indicators. Additional studies are necessary.

In conclusion, Peng’s Shengjing recipe improves the quality of sperm and shows effectiveness in patients with clinical asthenospermia due to kidney yang deficiency. The treatment was well tolerated, without obvious hepatorenal toxicity.

## References

[1] Jungwirth A , Diemer T , Kopa Z , Krausz C , Minhas S , Tournaye H. EAU Guidelines on Male Infertility. Arnhem: European Association of Urology; 2018: 324–332. 10.1016/j.eururo.2012.04.048 22591628

[2] Practice Committee of the American Society for Reproductive M. Diagnostic evaluation of the infertile male: a committee opinion. Fertil Steril 2015; 103: e18–e25. 25597249 10.1016/j.fertnstert.2014.12.103

[3] Fields E , Chard J , James D , Treasure T , Guideline Development G. Fertility (update): summary of NICE guidance. BMJ 2013; 346: f650. 23427132 10.1136/bmj.f650

[4] Smits RM , Mackenzie-Proctor R , Yazdani A , Stankiewicz MT , Jordan V , Showell MG. Antioxidants for male subfertility. Cochrane Database Syst Rev 2019; 3: CD007411. 30866036 10.1002/14651858.CD007411.pub4 PMC6416049

[5] Liu J , Dai Y , Li Y , Yuan E , Wang Q , Wang X , et al. A longitudinal study of semen quality among Chinese sperm donor candidates during the past 11 years. Sci Rep 2020; 10: 10771. 32612270 10.1038/s41598-020-67707-x PMC7329839

[6] Huang C , Li B , Xu K , Liu D , Hu J , Yang Y , et al. Decline in semen quality among 30,636 young Chinese men from 2001 to 2015. Fertil Steril 2017; 107: 83-8 e2. 10.1016/j.fertnstert.2016.09.035 27793371

[7] Zhou SH , Deng YF , Weng ZW , Weng HW , Liu ZD. Traditional Chinese Medicine as a remedy for male infertility: A review. World J Mens Health 2019; 37: 175–185. 30644235 10.5534/wjmh.180069 PMC6479084

[8] Pan X , Wang X , Wang X , Zhang W , Sun Z , Liang X , et al. Protective effects of new Wenshen Shengjing Decoction on cyclosporine-induced impairment of testosterone synthesis and spermatogenic apoptosis. Exp Ther Med 2018; 15: 813–821. 29399088 10.3892/etm.2017.5473 PMC5772751

[9] Xu DH , Wang LH , Mei XT , Li BJ , Lv JL , Xu SB. Protective effects of seahorse extracts in a rat castration and testosterone-induced benign prostatic hyperplasia model and mouse oligospermatism model. Environ Toxicol Pharmacol 2014; 37: 679–688. 24607683 10.1016/j.etap.2014.02.001

[10] Dong WW , Huang HL , Yang W , Liu J , Yu Y , Zhou SL , et al. Testis-specific Fank1 gene in knockdown mice produces oligospermia via apoptosis. Asian J Androl 2014; 16: 124–130. 24369145 10.4103/1008-682X.122592 PMC3901870

[11] Sun ZG , Lian F , Jiang KP , Zhang JW , Ma FM , Zhang N , et al. [Shengjing prescription improves semen parameters of oligoasthenozoospermia patients: efficacy and mechanism]. Zhonghua Nan Ke Xue 2012; 18: 764–767. 22934526

[12] Zhang YT , Yuan JL , Wang Y. The influence of Compound Xuanju Capsule on morphology and motility of the sperm of infertile male. Chin J Andrology 2009; 23: 52–54.

[13] Kong YJ , Wei WY. Treatment of 60 cases of male oligospermia and asthenospermia with compound Xuanju capsule. J Pract Trad Chin Med 2020; 36: 1278–1279.

[14] World Health Organization. World Health Organization laboratory manual for the Examination and processing of human semen. [Updated 2010; 2020 Aug 17]. Available from: https://apps.who.int/iris/handle/10665/44261

[15] Wang S. Advanced Textbook on Traditional Chinese Medicine and Pharmacology. Beijing: New World Press; 1996.

[16] Yang BC , Zhang CX , Yang J. Clinical observations on therapeutic effects of the modified shengjing zhongzi tang (see text) in patients with asthenospermia and oligozoospermia. J Tradit Chin Med 2011; 31: 192–194. 21977861 10.1016/s0254-6272(11)60040-x

[17] Song FW , Zhong WD. [Clinical efficacy of Shengjing capsule on patients with oligoasthenospermia]. Zhonghua Nan Ke Xue 2009; 15: 762–764. 19852282

[18] Guo J , Song CS , Geng Q. [Clinical observation on treatment of oligospermia and asthenospermia with Liuwu Shengjing Decoction]. Zhongguo Zhong Xi Yi Jie He Za Zhi. 2007; 27: 986–988. 18173142

[19] Zhang HQ , Zhao HX , Zhang AJ. [Male infertility with severe oligospermatism and azoospermia treated by Bushen Shengjing Decoction combined with intracytoplasmic sperm injection]. Zhongguo Zhong Xi Yi Jie He Za Zhi 2007; 27: 972–975. 18173138

[20] Chen RA , Wen H. [Clinical study on treatment of male infertility with shengjing pill]. Zhongguo Zhong Xi Yi Jie He Za Zhi 1995; 15: 205–208. 7647540

[21] Yue GP , Chen Q , Dai N. [Experimental study on effect of bushen shengjing decoction on kidney yang and testicular dysfunction in rats]. Zhongguo Zhong Xi Yi Jie He Za Zhi 1997; 17: 289–291. 9863114

[22] Liang XY , Liu XQ , Ding YB , Chen XM , Wang YX. [Genetic imprinted gene PEG10 expression in deciduas from inevitable abortion]. Yi Chuan 2008; 30: 735–740. 18550496 10.3724/sp.j.1005.2008.00735

[23] Li JT , Qu XW , Zhang SW , Li ZS , Zhang PH. [Effects of Yishen Shengjing Capsules on semen quality and gonadal hormone levels in rats with dibutyl phthalate-induced reproductive function injury]. Zhonghua Nan Ke Xue 2016; 22: 1110–1115. 29282917

[24] Yang AM , Li YQ , Li HS , Song CS , Ren Y , Yin JX. [Affection of Bushen Shengjing pill on NO and NOS in testicle and ante-oxidization of rat with spermatogenic cell injury]. Zhongguo Zhong Yao Za Zhi 2006; 31: 904–906. 17048630

[25] Wang J , Zhao S , Luo L , Liu Y , Li E , Zhu Z , et al. Shengjing Capsule Improves Spermatogenesis through Upregulating Integrin alpha6/beta1 in the NOA Rats. Evid Based Complement Alternat Med 2019; 2019: 8494567. 31534468 10.1155/2019/8494567 PMC6724431

[26] Merc V , Frolikova M , Komrskova K. Role of Integrins in Sperm Activation and Fertilization. Int J Mol Sci 2021; 22: 11809. 34769240 10.3390/ijms222111809 PMC8584121

